# Battling *Salmonella* enteritidis infections: integrating proteomics and in vivo assessment of *Galla Chinensis* tannic acid

**DOI:** 10.1186/s12917-024-04036-5

**Published:** 2024-05-07

**Authors:** Yuzhang Yan, Xiaohong Zheng, Xueqin Wu, Ling Wang, Jiongjie He, Baocheng Hao, Tingjun Hu, Shengyi Wang, Dongan Cui

**Affiliations:** 1 Key Laboratory of New Animal Drug Project, Key Laboratory of Veterinary Pharmaceutical Development, Ministry of Agriculture and Rural Affairs, Lanzhou Institute of Husbandry and Pharmaceutical Sciences of Chinese Academy of Agriculture Sciences, No. 335, Jiangouyan Street, Qilihe District, Lanzhou, Gansu Province 730050 P.R. China; 2 College of Animal Science and Technology, Guangxi University, Nanning, 530005 China

**Keywords:** Proteomics, Antibacterial activity, *Galla Chinensis* tannic acid, *Salmonella enterica* serovar Enteritidis

## Abstract

*Salmonella* infections pose a significant threat to animal and human health. Phytochemicals present a potential alternative treatment. *Galla chinensis* tannic acid (GCTA), a hydrolyzable polyphenolic compound, inhibits bacterial growth and demonstrates potential as an alternative or supplement to antibiotics to prevent *Salmonella* infections. However, little is known about the antimicrobial mechanism of GCTA against *Salmonella*. Here, we revealed 456 differentially expressed proteins upon GCTA treatment, impacting pathways related to DNA replication, repair, genomic stability, cell wall biogenesis, and lipid metabolism using TMT-labeled proteomic analysis. TEM analysis suggested altered bacterial morphology and structure post-treatment. A *Salmonella-*infected-mouse model indicated that GCTA administration improved inflammatory markers, alleviated intestinal histopathological alterations, and reduced *Salmonella enterica* serovar Enteritidis (*S*. Enteritidis) colonization in the liver and spleen of *Salmonella*-infected mice. The LD_50_ of GCTA was 4100 mg/kg with an oral single dose, vastly exceeding the therapeutic dose. Thus, GCTA exhibited antibacterial and anti-infective activity against *S*. Enteritidis. Our results provided insight into the molecular mechanisms of these antibacterial effects, and highlights the potential of GCTA as an alternative to antibiotics.

**Supplementary Information:**

The online version contains supplementary material available at 10.1186/s12917-024-04036-5.

## Introduction

*Salmonella* spp. encompasses a diverse group of bacteria that can cause infection in a variety of hosts [1]. These *Salmonella* spp. constitute significant foodborne pathogens, responsible for inducing gastroenteritis and bacteremia in human hosts [2]. Of particular concern is *Salmonella enterica* serovar Enteritidis, which poses a substantial public health threat as a primary causative agent of foodborne diseases, with an estimated annual mortality rate exceeding 300,000 [3, 4]. The recent emergence of multidrug-resistant *Salmonella* spp. poses a serious threat to global health, especially considering the potential transfer of antibiotic-resistant strains from livestock to humans [5]. Thus, the control of *Salmonella* remains both a therapeutic challenge and a goal under the One Health initiative [1], and there is a need to develop alternatives to traditional antibiotics.

Compounds derived from traditional herbal medicines have emerged as potential substitutes, offering antimicrobial properties with a reduced likelihood of inducing antibiotic resistance [6]. These include alkaloids, terpenoids, phenolic compounds and other phytochemicals [7]. Tannic acid is a hydrolysable polyphenol commonly found in plants that induces a variety of biological effects including antimicrobial [8] and anti-diarrheal activity [9] and the ability to regulate microbial homeostasis [10]. At the appropriate dose, tannic acid may improve gut health and growth performance without producing undesirable antinutritional effects [11–13]. Due to its strong antimicrobial activity [14], tannic acid represents a potential alternative to conventional antibiotics.

*Galla chinensis* is a component of the gall produced by the aphid *Melaphis chinensis*, and it can be divided by shape into two categories: *jiaobei* and *dubei.* As a Chinese herb, it is employed in the treatment of various ailments, including diarrhea, constipation, bleeding, cough, vomiting, and sweating [15]. Phytochemical investigations have demonstrated that that *Galla chinensis* is rich in gallotannins. Of these, *Galla chinensis* tannic acid (GCTA) is a hydrolysable tannin representing at least 70% of its total weight [16]. GCTA has been found to inhibit bacterial growth [17], modulate host immune responses [18], improve intestinal barrier function [11] and influence the gut microbiota [19]. GCTA has demonstrated anti-virulence by targeting the type III secretion system against *Salmonella* [20]. GCTA thus represents a candidate therapeutic in *Salmonella* infections.

Here, we explore the inhibitory effect of GCTA toward *S.* Enteritidis ATCC13076 by evaluating the MIC using the broth microdilution method and invasion assay. We further employ proteomic analysis and transmission electron microscopy (TEM) to determine the molecular mode of GCTA action against *S*. Enteritidis. Finally, we evaluate GCTA against *Salmonella* challenge in a mouse model, and comprehensively analyze its in vivo antibacterial effects. Collectively, these findings provide a new strategy for *Salmonella* treatment.

## Materials and methods

### GCTA preparation

*Galla chinensis* (GC) was supplied by the Fuxinghou biomedicine Technology Co., Ltd. (Gansu, PR China). After pre-processing by washing and drying, GC was pulverized to a particle size of 250–425 μm with 40–60 mesh (Jingding, Zhejiang, China). We weighed a total of 100.0 g of lyophilized GC and then refluxed it three times over 1.5 h with water (1:8, w/v). The resulting filtrates were concentrated at 60 °C by a rotary evaporator (RV8, IKA, Germany) and freeze-dried to yield the extract, weighing 82.2 g. The quantity was detected according to the quality standard of GC in the Chinese pharmacopoeia [21], and the content of Gallic acid and the tannins reached 716.8 mg/g and 536.0 mg/g, respectively.

### Chromatographic fingerprint of GCTA by HPLC

HPLC fingerprinting analysis was conducted using a Waters e2695 HPLC system (Waters Corporation, Milford, USA), consisting of a Waters2996 PDA detector, a quota pump, and Waters Empower2 Chromatographic software. A column of Agilent ZORBAX SB C_18_ (5 μm, 250 × 4.6 mm) was used for separation at 25 °C. We used HPLC with gradient elution at 0.8 mL per minute and injection volumes of 10 µL. Two solvents were used for the mobile phase: (A) 100% methanol and (B) 0.2% formic acid as indicated in Table 1. Fingerprints were obtained at a wavelength of 280 nm, with Gallic acid serving as a set of multiple references to align the retention time of the chromatograms.

**Table 1:** Gradient elution of mobile phase

Time (min)	Mobile phase A (%)	Mobile phase B (%)
0	5	95
20	20.8	79.2
30	22.7	77.3
35	24.5	75.5
40–55	28.7	71.3
80	31	69
85	95	5
85.5	5	95

### Bacteria strains and culture conditions

*S.* Enteritidis strain ATCC13076, obtained from the American Type Culture Collection (ATCC; Manassas, Virginia), was stored at − 80 °C with 40% glycerol. A loopful of bacterial culture was plated onto Luria-Bertani (LB; Huankai Biology, Guangzhou, China) medium and incubated at 37 °C overnight. Single colonies were then inoculated into 5 mL LB broth (37 °C, 225 rpm, 6–8 h) to late log phase. For experiments involving animals, cultures were grown in LB broth (37 °C, 225 rpm, 18 h) to attain stationary phase. Bacteria concentrations were adjusted in sterile pharmaceutical grade saline (SPGS) based on OD_600_. Inoculum concentrations were verified by plating tenfold serial dilutions on LB plates for counting of colony forming units (CFUs).

### Minimal inhibitory concentration (MIC), minimal bactericidal concentration (MBC) and time-kill curve of GCTA against *S.* Enteritidis

Mueller-Hinton broth (MHB, Huankai Biology, Guangzhou, China) microdilution assays were used to test the MIC and MBC of GCTA against *S.* Enteritidis according to CLSI M07 recommendations [22]. Bacterial suspensions were adjusted to 0.5 McFarland (1 × 10^8^ CFU/mL). Samples were diluted by tenfold serial dilutions to 5 × 10^5^ CFU/mL; final concentration gradients of GCTA were 44.8, 22.4, 11.2, 5.6, 2.8, 1.4, 0.7, 0.35, 0.175 and 0 mg/mL. MIC, denoting the lowest GCTA concentration inhibiting bacterial growth, was determined, while MBC, the lowest GCTA concentration without a single colony on MHB agar after 18 h at 37 °C, was also assessed. Additionally, MIC and MBC of GCTA were measured against various concentrations of *S*. Enteritidis (10^3^, 10^5^, and 10^8^ CFU/mL).

*S.* Enteritidis was incubated in MHB with GCTA based upon MIC (final concentrations of 1 MIC, 2 MIC, 4 MIC or 8 MIC). Samples were collected at specific time intervals (0, 0.5, 1, 2, 3, 4, 6, 8, 10, 14, 16 h) and subsequently cultured on blood agar (Huankai Biology Co., Ltd., Guangzhou, China) for enumeration. The time-kill curves were generated by plotting the logarithm of CFUs versus time.

### Cell culture

Caco-2 cells were acquired from the National Collection of Authenticated Cell Cultures (NCACC, Shanghai, China) and were cultivated in Minimum Essential Medium (MEM, Gibco, USA). Anderson RAW 264.7 cells (NCACC, Shanghai, China) were cultivated in Dulbecco’s Modified Eagle Medium (DMEM, Gibco, China) supplemented with 10% fetal bovine serum (FBS) and 1% penicillin-streptomycin. Both cell lines were maintained at a temperature of 37 °C in a 5% CO_2_ atmosphere, with media changes performed every 2 days.

### Adhesion and invasion assay

As per Mechesso et al.’s gentamicin protection assay [23], Caco-2 and RAW 264.7 cells (2 × 10^5^/mL) were cultured in 24-well plates using MEM or DMEM. After a 48-hour incubation period for Caco-2 cells and 12-hour for RAW 264.7 cells, cultures were exposed to varying concentrations of GCTA (0–10 µM) over 2 h. Subsequently, 8-hour *S*. Enteritidis cultures were prepared, centrifuged (3,000 rpm, 10 min), and washed twice with 1×SPGS. The infection of cells occurred at a multiplicity of infection of 1:100. After incubation at 37 °C for 1 h, removal of supernatant, and three washes with 1×SPGS, cell lysis with 500 µL 1% Triton X-100 ensued. Plating tenfold serial dilutions on LB plates to quantify the total number of adherent bacteria (CFU/mL).

For invasion assay, GCTA-pretreated and *S.* Enteritidis-infected cells were incubated 1 h following the same protocol as the adhesion assay. After three washes with 1×SPGS, cells were incubated for 1 h in medium supplemented with gentamicin (100 µg/mL). The procedure for cell lysis and quantification of intracellular bacteria (CFU/mL) was similar to the adhesion assay. Both invasion and adhesion experiments were repeated three times.

### TMT-labeled quantitative proteomic analysis

*S.* Enteritidis (1 × 10^8^ CFU/mL) cultured with 1 MIC of GCTA in MHB (37 °C, 1 h) were subjected to centrifugation, washed with SPGS, and flash-frozen in liquid nitrogen. Samples were subjected to lysis using SDT lysis buffer (4% SDS, 100 mM Tris-HCl, pH 7.6), followed by homogenization, sonication, and boiling. The resulting supernatant was quantified using the BCA Protein Assay Kit (Beyotime, Shanghai, China) and then subjected to SDS-PAGE for confirmation of purity and extraction efficiency.

Subsequently, 200 µg protein samples were treated with dithiothreitol (DTT, Sigma, USA), followed by centrifugation and washing. Iodoacetamide (Sigma, USA) treatment, trypsin digestion (4 µg Trypsin in 40 µL 0.1 M TEAB buffer, Promega, USA), and sequential centrifugation were performed to obtain labeled peptides for analysis. HPLC separation and analysis with a Q Exactive plus mass spectrometer (Thermo Fisher, USA) ensued.

The functional annotation of the proteins identified in this study was performed using various tools and databases, including WoLF PSORT, Gene Ontology (GO) annotation, Evolutionary Genealogy of Genes (EggNOG) database, and Kyoto Encyclopedia of Genes and Genomes (KEGG) database. To identify differentially expressed proteins (DEPs) in the GCTA-treated group compared to the untreated control group, univariate analysis was employed. Proteins with a fold change (FC) > 1.2 and a p-value < 0.05, determined by t-test, were considered differentially expressed.

### GCTA treatment and preparation for TEM

The impact of GCTA on the morphology and structure of *S.* Enteritidis was investigated using TEM (Hitachi H7700, Tokyo, Japan). *S.* Enteritidis cultures (1 × 10^8^ CFU/mL) were treated with 1 MIC of GCTA for 2 h. Then the culture was centrifuged, and the resulting precipitates were immersed in fixative (2.5% glutaraldehyde in 0.1 M phosphate buffer, Servicebio, China) at 4 °C overnight. Precipitates underwent triple rinsing with 0.1 M phosphoric acid, followed by pre-embedded in agarose and fixation with 1% OsO4 in 0.1 M phosphate buffer (PB, pH 7.4) for 2 h. After dehydration, the samples were double-stained with 2% uranium acetate and 2.6% lead citrate for TEM analysis. Control samples were prepared without GCTA.

### Animals

BALB/c SPF mice (females, 8 weeks old, 18–22 g) were obtained from the Center of Experimental Animals of Lanzhou Veterinary Research Institute (Gansu, China). The mice were kept in a controlled environment with specific pathogen-free (SPF) barrier conditions, in a room with regulated temperature and alternating 12-hour light-dark cycles. Unless otherwise stated, mice had *ad libitum* access to food and water, and those that were infected were subjected to daily monitoring to detect any indications of clinical illness. All mice were humanely euthanized using carbon dioxide asphyxiation followed by cervical dislocation, in accordance with approved ethical guidelines in the present study.

### Oral acute toxicity assay of GCTA in mice by up-and-down procedure (UDP)

The acute oral toxicity of GCTA in mice was evaluated using the UDP following Organization for Economic Cooperation and Development Guideline No. 425 [24]. According to previous literature [15, 39] and our pilot data (data not shown), the estimated LD_50_ was 4000 mg/kg. The dosages were calculated by AOT425StatPgm based on an assumed sigma of 0.125. The initial dosage of 3100 mg/kg resulted in the manifestation of poisoning symptoms within a 24-hour period. Subsequent dose adjustments were determined based on the survival outcome of the preceding dose. The experimental sequence continued until the standard stopping rules were met. Post-dosing, survival and death were recorded, and mice surviving for 14 days were observed daily.

### In vivo experimental infection with *S.* Enteritidis

Mice were randomized into 5 groups (*n* = 5): Control, *S*. Enteritidis + Vehicle, and *S*. Enteritidis + GCTA (50, 100 and 200 mg/kg). Prior to infection, animals received streptomycin (100 mg/kg) for 3 consecutive days and were then orally gavaged with 0.1 mL of SPGS, with or without 1 × 10^8^ CFU ATCC13076, for 2 consecutive days. Six hours post final infection, mice were administered either GCTA or SPGS at doses of 50, 100, or 200 mg/kg once daily for five consecutive days. Mice were euthanized 6 h after the final treatment.

#### Blood sample analysis

Following anesthesia (Pentobarbital sodium, 50 mg/kg BW dose intraperitoneal injection), blood was collected via cardiac puncture and placed into non-heparinized tubes (Yuli Medical Insrtument, Jiangsu, China) for separation of serum. Serum concentrations of tumor necrosis factor-α (TNF-α), interleukin-1β (IL-1β), interleukin-6 (IL-6), and interleukin-10 (IL-10) were assessed using ELISA kits (Shanghai Enzyme-Linked Biotechnology, Shanghai, China) in accordance with the manufacturer’s instructions.

####  H&E staining and histochemistry

The distal ileum (  6-cm length above the cecum) was collected and fixed in 4% paraformaldehyde for a minimum of 1 week. Following routine paraffin embedding and fixation, the tissue was subjected to Hematoxylin-eosin (H&E) staining to assess the severity of intestinal inflammation, mucosal injury extent, villi and crypt damage. Villus height (VH) and crypt depth (CD) were measured using the methodology outlined by Ekim et al. [25].

#### Detection of bacterial load in liver and spleen

According to the procedures described by Khan and Chousalkar [26], aseptic collection of liver and spleen sections was performed, placing then into 1.5 mL Safe-Lock Eppendorf tubes (Eppendorf, Germany) with stainless steel beads (0.5–2.0 mm) and 500 µL SPGS. Following weighing, each tissue sample underwent homogenization using a bullet blender (Next Advance, USA) at full speed for 5–10 min. A 100 µL aliquot of the original tissue homogenates or tenfold serial diluted samples was plated onto Xylose Lysine Deoxycholate Agar (XLD Agar, Huankai Biology, China) and incubated overnight at 37 °C to quantify the bacterial load.

### Statistical analysis

One-way ANOVA using SPSS 22.0 (SPSS Inc., Chicago, IL, United States) was subjected to MIC, MBC, bodyweights, serum levels of inflammatory markers, histomorphological parameters and *Salmonella* counts. Statistical significance was declared at *p* < 0.05. GraphPad Prism v 8.0 was used to generate graphs (GraphPad Software, Inc., San Diego, USA), and the data were expressed as the mean ± standard error of the mean (SEM).

## Results

### Chromatographic fingerprint of GCTA

**Fig. 1:**
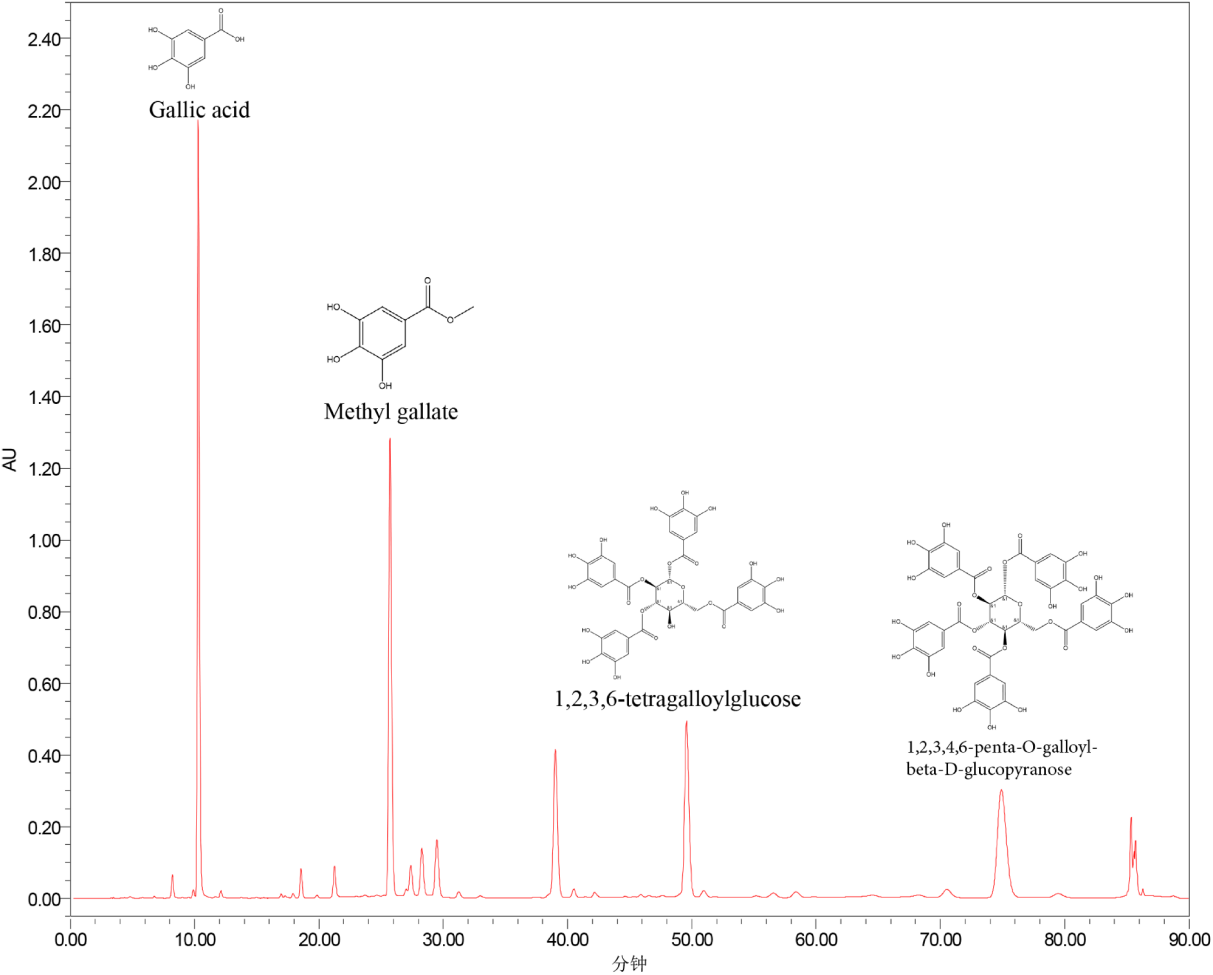
Representative HPLC chromatograms of GCTA. 2: Gallic acid (S); 4: Methyl gallate; 7: 1,2,3,6-tetra-O-galloyl-*β*-D-glucose; 8: 1,2,3,4,6-penta-O-galloyl-β-D-glucose. Chemical structure of the remaining compounds (1, 3, 5, 6) were uncertain

**Fig. 2:**
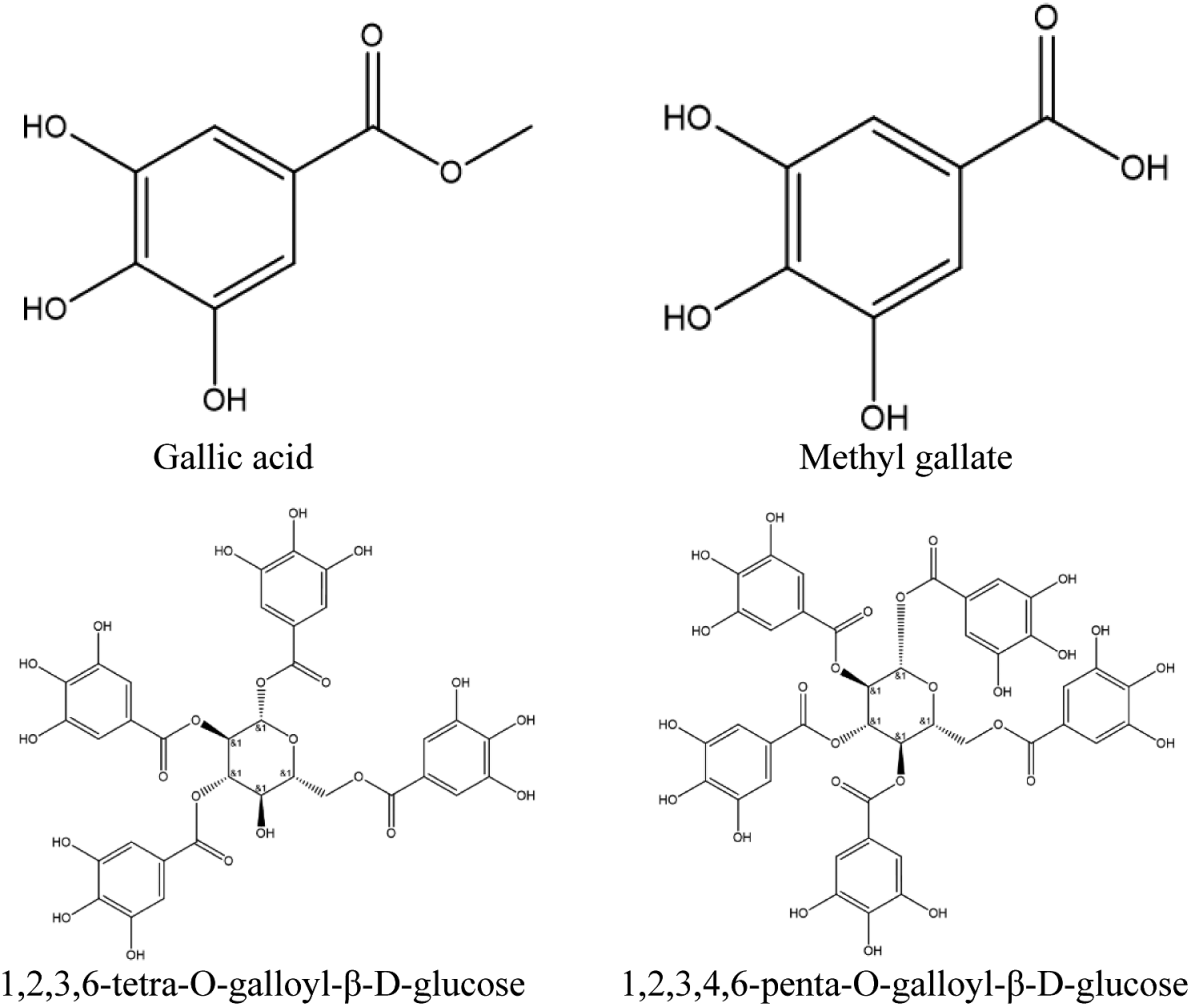
Structures of the four analytes

Figure 1 shows a representative chromatogram for GCTA. According to the relative retention time of the reference chromatogram, the peaks 4, 6 and 7 were recognized as 1,2,3,6-tetra-O-galloyl-β-D-glucose and 1,2,4,6-tetra-O-galloyl-*β*-D-glucose; 1,2,3,4,6-penta-O-galloyl-β-D-glucose. The structures of the four analytes are shown in Fig. 2.

### Antibacterial effect of GCTA on *S.* Enteritidis

**Fig. 3:**
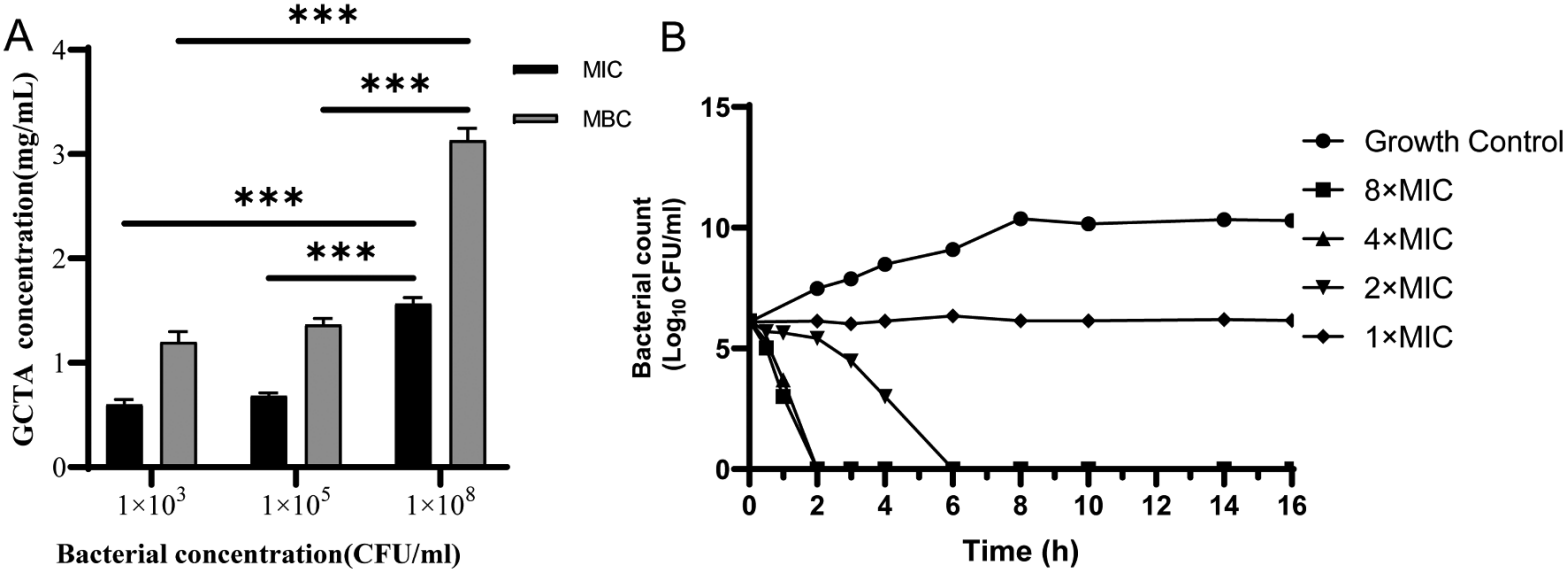
Antibacterial effect of GCTA against *S.* Enteritidis. (A) The effect of different concentrations of *S.* Enteritidis on MIC and MBC of GCTA. (B) The time-bactericidal curve of GCTA against *S.* Enteritidis. Data are represented as mean ± SEM of three independent experiments. *p ≤ 0.05, **p ≤ 0.01, ***p ≤ 0.001)

The MIC of GCTA against *S.* Enteritidis ATCC13076 (5 × 10^5^ CFU/mL) was 0.72 ± 0.03 mg/mL and the MBC was 1.43 ± 0.06 mg/mL (Fig. S1). The effect of GCTA was evaluated against different concentrations of ATCC13076 (Fig. 3A). The MIC and MBC significantly increased with the rise in bacterial concentration to 10^8^ CFU/mL (*p* < 0.001). The relationship between the concentration of GCTA and its bactericidal effect was evaluated using time-bactericidal curves (Fig. 3B). At 1 MIC of GCTA, there was no significant change in bacterial count observed within 16 h. However, at 2 MIC of GCTA, the bacterial count significantly decreased after 2 h of incubation, and after 6 h there were almost no bacteria left (Fig. 3B). At 4 MIC and 8 MIC of GCTA, all bacteria were killed within 2 h.

### Impacts of GCTA on *S.* Enteritidis adhesion and invasion

**Fig. 4:**
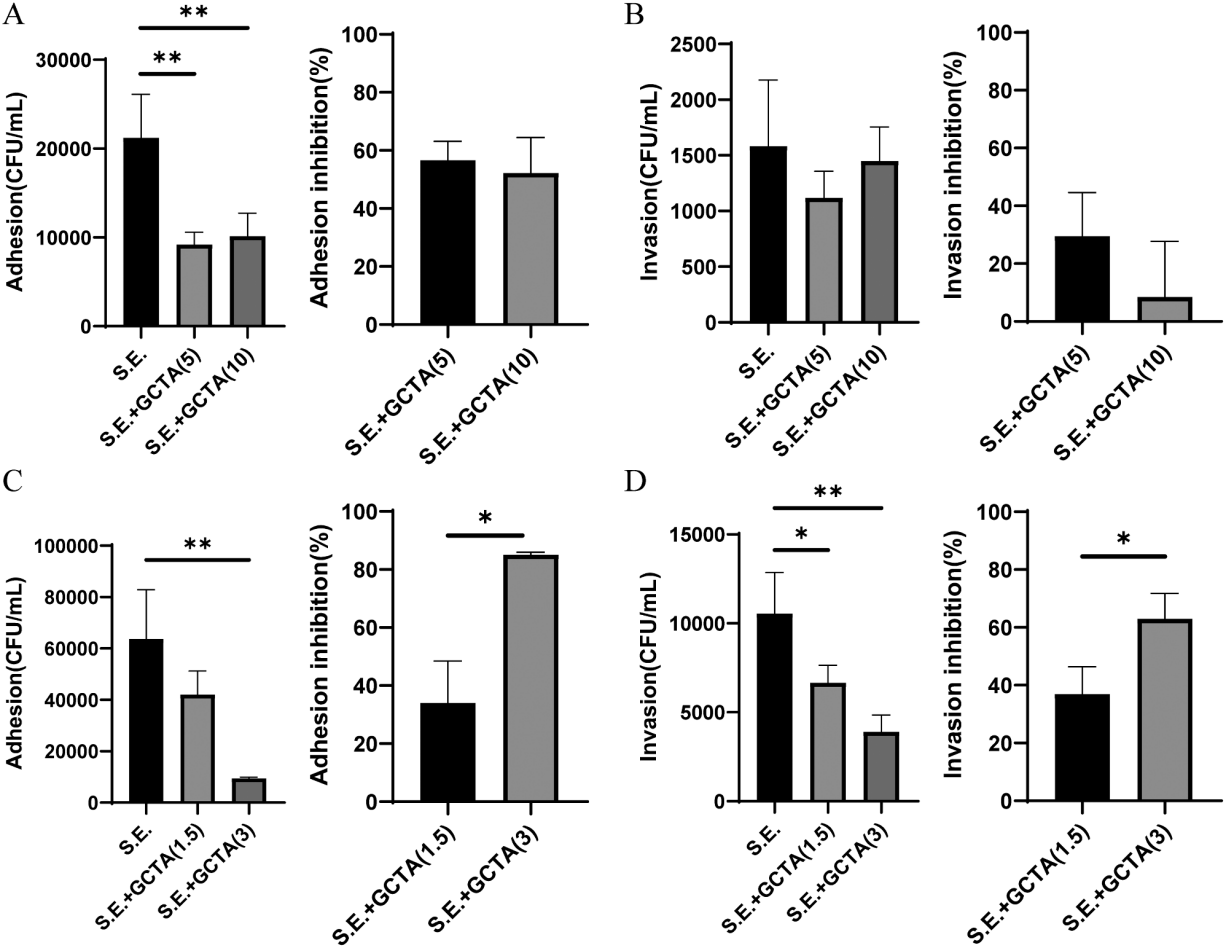
Inhibitory effects of GCTA on the adhesion and invasion of *S*. Enteritidis to cells in vitro. (**A**) The impact of 5 or 10 µM GCTA on adhesion (left) and the percentage inhibition of adhesion (right) of *S*. Enteritidis to Caco-2 intestinal epithelial cells. (**B**) The impact of 5 or 10 µM GCTA on invasion (left) and the percentage inhibition (right) of invasion of *S*. Enteritidis to Caco-2 intestinal epithelial cells. (**C**) The impact of 1.5 or 3 µM GCTA on adhesion (left) and the percentage inhibition (right) of adhesion of *S*. Enteritidis to RAW 264.7 macrophage cells. (**D**) The impact of 1.5 or 3 µM GCTA on invasion (left) and the percentage inhibition (right) of invasion of *S*. Enteritidis to RAW 264.7 macrophage cells. All data are presented as the mean ± SEM of three independent experiments

Preincubation of Caco-2 cells with concentrations of 5 and 10 µM of GCTA led to a two-fold decrease in the adhesion of *S*. Enteritidis compared to the controls (Fig. 4A). However, neither concentration of GCTA reduced the invasion of *S.* Enteritidis into Caco-2 cells (Fig. 4B). Furthermore, 3 µM GCTA significantly reduced both the adhesion (Figs. 4C and 85.2%, *p* < 0.01) and invasion (Figs. 4D and 62.9%, *p* < 0.01) of *S.* Enteritidis into RAW 264.7 cells. Contrary to Caco-2 cells, when RAW 264.7 cells were treated with 1.5 µM of GCTA, there was a significant reduction (36.9%, *p* < 0.05) in the invasion of *S.* Enteritidis (Fig. 4D).

### Protein profiling of GCTA-treated *S.* Enteritidis

**Fig. 5:**
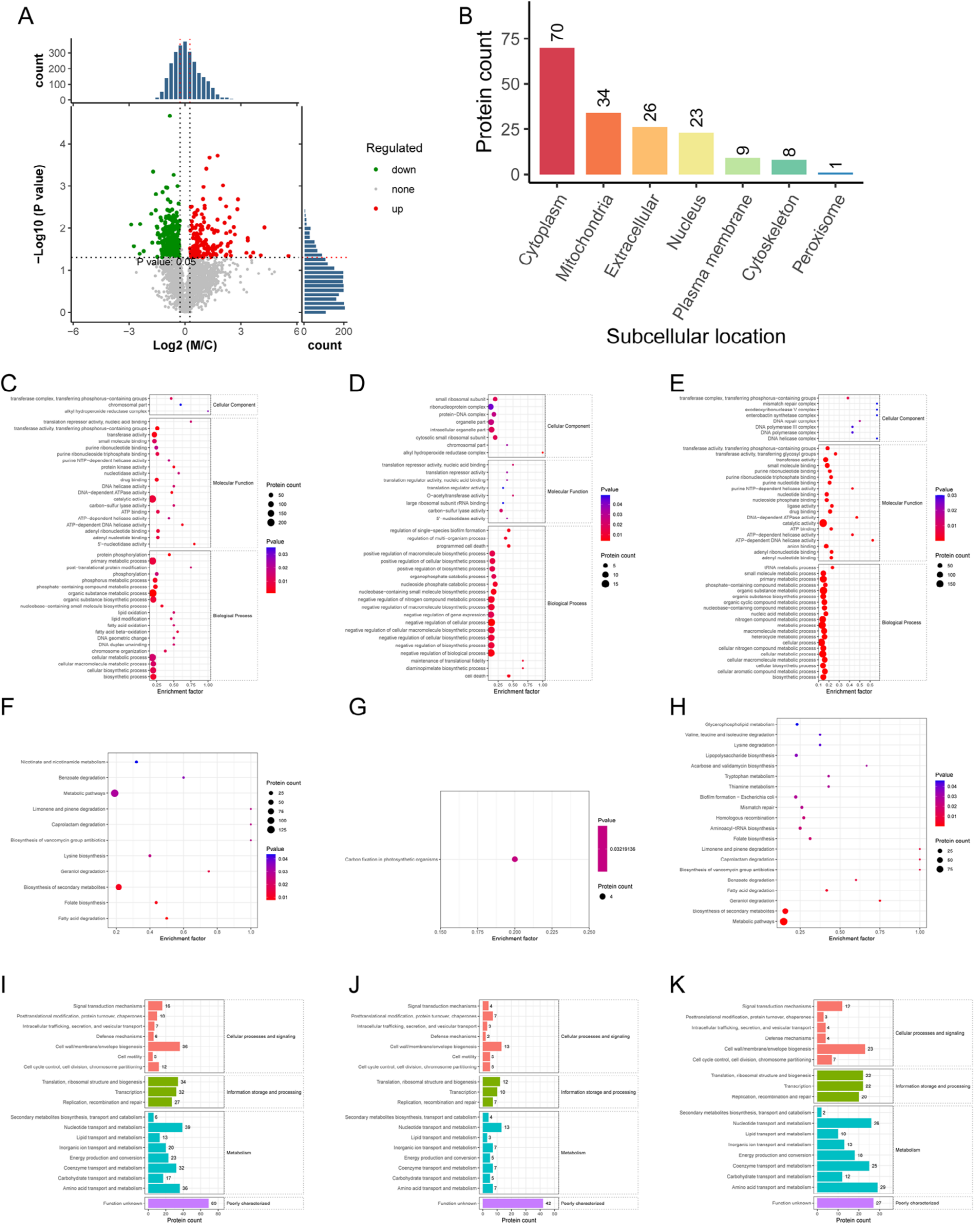
Expression and functional analysis of proteins identified by TMT-labeled quantitative proteomics in GCTA-treated *S.* Enteritidis. (**A**) The volcano map of DEPs. The abscissa denotes the ratios of DEPs in GCTA-treated, M, versus untreated, C, *S.* Enteritidis groups; the ordinate represents the *P*-values between the two groups. (**B**) Subcellular location of differential proteins. (**C**-**E**) GO enrichment cluster analysis of the differential proteins. (**D**) Upregulated DEPs following GCTA treatment. (**E**) Downregulated DEPs following GCTA treatment. (**F**-**H**) KEGG pathway clustering heat map of the differential proteins. (**G**) Upregulated proteins following GCTA treatment (**H**) Downregulated proteins following GCTA treatment. (**I**-**K**) EggNOG enrichment histogram of differential proteins. (**J**) Upregulated proteins following GCTA treatment (**K**) Downregulated proteins following GCTA treatment. Abbreviations: GO, Gene Ontology; KEGG, Kyoto Encyclopedia of Genes and Genomes; EggNOG, Non-supervised Orthologous; TMT, Tandem Mass Tag

TMT isotope labeling identified 456 DEPs in GCTA-treated *S*. Enteritidis cultures (Fig. 5A; FC > 1.2, *p* < 0.05). Compared to control, 171 proteins were up-regulated and 285 proteins were down-regulated in GCTA-treated *S*. Enteritidis. Among them, the DEPs annotated to genomic stability, cell wall/membrane/envelope biogenesis, lipid metabolism identified, glycerophospholipid metabolism identified and lipopolysaccharide biosynthesis attracted further attention (Tables S1-S5).

### Analysis of DEP pathways and functions of differentially regulated proteins

The prediction analysis of subcellular localization showed that the 456 DEPs were distributed among the cytoplasm (153), mitochondria (109), nucleus (64), plasma membrane (44), extracellular (41) and peroxisome (2) (Fig. 5B).

GO enrichment analysis demonstrated enrichment in post-translational protein modification, lipid oxidation, fatty acid oxidation, DNA geometric change and DNA duplex unwinding comprised over 50% of DEPs (Fig. 5C). Among these, proteins involved in alkyl hydroperoxide reductase complex, translation repressor activity, nucleic acid binding, O − acetyltransferase activity and lipopolysaccharide biosynthesis were upregulated (Fig. 5D), whereas the expression of DEPs associated with ATP-dependent DNA helicase activity was diminished in the GCTA-treated group compared to control (Fig. 5E).

KEGG enrichment analysis revealed significant differences in the expression of proteins annotated to limonene and pinene degradation, caprolactam degradation and biosynthesis of vancomycin group antibiotics (Fig. 5F). Enrichment of down-regulated DEPs involved in acarbose and validamycin biosynthesis, benzoate degradation and geraniol degradation met or exceeded 50% (Fig. 5G, H). Functional classification with EggNOG statistical analysis (Fig. 5I) showed that many DEPs were involved in cell wall, membrane, and envelope biogenesis, translation, ribosomal structure and biogenesis, transcription, and in nucleotide, coenzyme and amino acid transport and metabolism (Fig. 5J, K).

### GCTA altered morphology and structure of *S.* Enteritidis

**Fig. 6:**
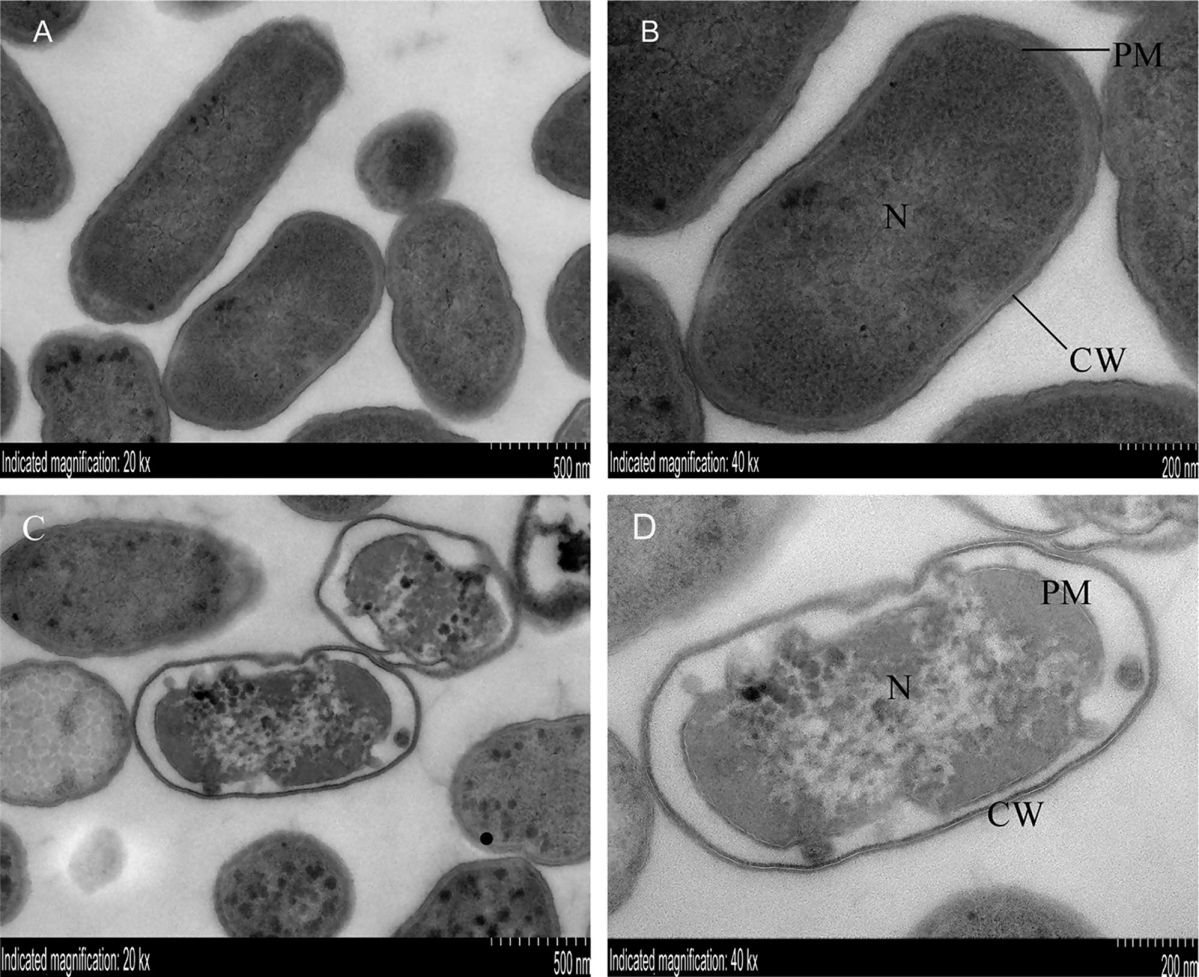
Alterations in morphology and structure of GCTA treated *S*. Enteritidis (**A**, **B**) Morphology of untreated *S*. Enteritidis. (**C**, **D**) Morphological changes in *S*. Enteritidis following 2 h GCTA treatment. In Fig. 6D, the cell wall (CW) was uniform, intact, continuous and locally invaginated; the cell membrane (PM) was atrophied, the local structure is vague and damaged, and the plasma wall was separated obviously; the filamentous structure of the nucleoid region (N) was sparse; and the plasmid (P) was scattered in the distributed cytoplasm

After GCTA treatment for 2 h, bacteria were collected for TEM analysis. Untreated bacteria showed intact bacilliform morphology with evenly distributed nucleoplasm (Fig. 6A, B), whereas alterations in the GCTA-treated group (Fig. 6C, D) included disruption of cell wall integrity, continuity, uniform thickness and local invagination; the cell membrane was crumpled, along with conspicuous plasmolysis and sparse filamented texture in the nuclear region.

### Acute oral toxicity of GCTA

To obtain the LD_50_ of GCTA in BALB/c mice, doses and intervals were designed by AOT425StatPgm. After acclimatization, mice were dosed with GCTA according to Table 2, and the LD_50_ of GCTA was 4100 mg/kg, 95% CI [3795, 4900].

**Table 2:** Lethality and signs of toxicity of mice administrated with GCTA by UDP

Seq.	Dosage (mg/kg)	Short-term outcome	Symptoms	Pathology
1	3100	O	Apparently normal	No discernible alterations observed in organs and tissues
2	4100	O	Reduced activity, recovered after 4.5 h	No discernible alterations observed in organs and tissues
3	5000	X	Lethargy, weakness, off feed, death in 3 days	Colonic constipation
4	4100	O	Reduced activity, recovered after 6 h	No discernible alterations observed in organs and tissues
5	5000	X	Lethargy, off feed, death in 5 days	Colonic constipation
6	4100	X	Reduced activity, dead after 10 days	Intestinal mucosa edema, flatulence
7	3100	O	Apparently normal	No discernible alterations observed in organs and tissues
8	4100	O	Reduced activity, recovered after 4 h	No discernible alterations observed in organs and tissues
9	5000	X	Lethargy, off feed, death in 4.5 days	Colonic constipation

*Note* The sequence of outcomes: X, death; O, survival

### GCTA mitigates enteritis in *S.* Enteritidis-infected mice

**Fig. 7:**
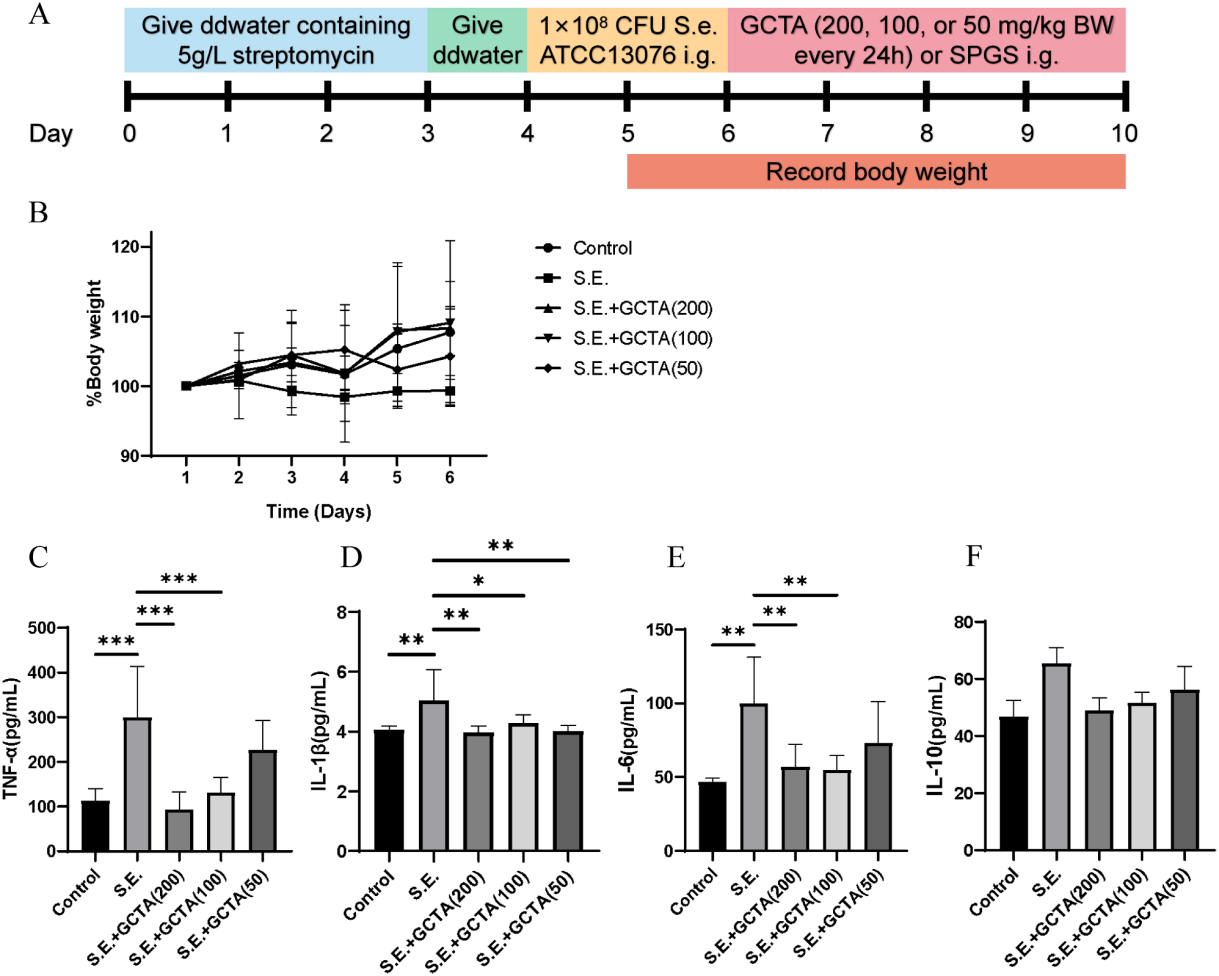
Treatment with GCTA mitigated enteritis in *S*. Enteritidis-infected mice. (**A**) Schematic of the dose regimen for the establishment of infection and GCTA treatment. (**B**) Percent change in body weight from initiation of infection (Day 5) for treated and untreated BALB/c mice. (**C**-**F**) Serum levels of TNF-α, IL-1β, IL-6 and IL-10 were measured using standard ELISA methods. S.E., *S*. Enteritidis; *n* = 5 per group; data are represented as mean ± SEM. (*p ≤ 0.05, **p ≤ 0.01, ***p ≤ 0.001)

The therapeutic effect of GCTA against *S.* Enteritidis was evaluated in BALB/c mice. After oral infection with *S.* Enteritidis for 2 days, mice were treated with GCTA (50, 100, or 200 mg/kg) for 5 days (Fig. 7A). Body weight comparison showed that *S.* Enteritidis infection reduced body weight and this was ameliorated by treatment, but the trend did not reach significance (Fig. 7B).

### GCTA modulates inflammatory markers

Serum of mice treated with 100 or 200 mg/kg GCTA had significantly reduced TNF-α, IL-1β and IL-6, hallmarks of *S.* Enteritidis infections (Fig. 7C-E). There was no significant change in serum IL-10 following treatment (Fig. 7F).

### GCTA resolved intestinal histopathologic changes

**Fig. 8:**
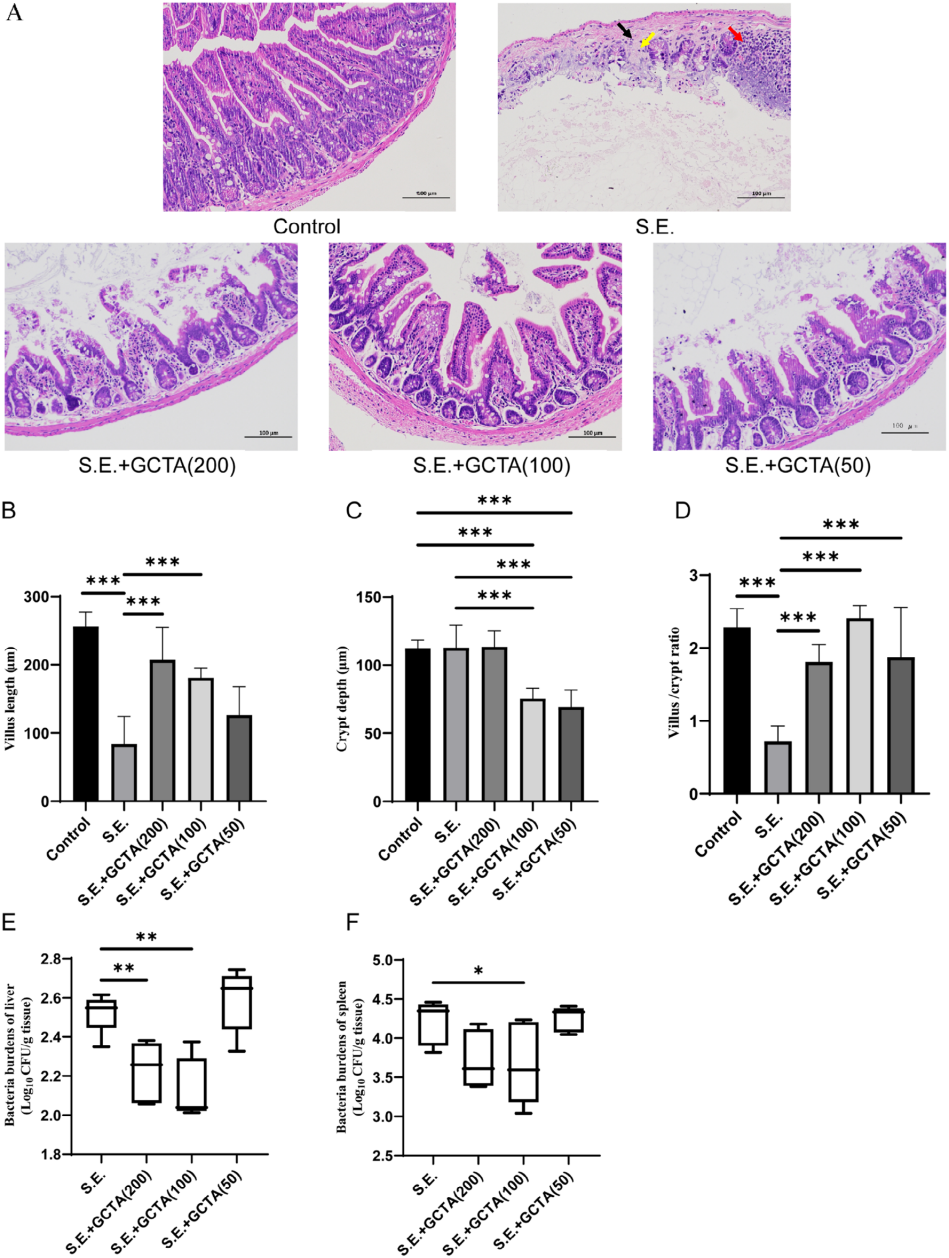
Treatment with GCTA improved intestinal histopathologic changes and inhibited *S*. Enteritidis colonization. (**A**) Effect of GCTA on histomorphological parameters of ileum of mice challenged with *S.* Enteritidis. The histological examination revealed a disruption of the mucosal epithelial integrity, characterized by necrosis and disintegration of the intestinal glands within the lamina propria. These areas were supplanted by proliferative connective tissue (indicated by a black arrow), accompanied by focal lymphocytic infiltration (denoted by a yellow arrow), and involvement of the regional lymph nodes (highlighted with a red arrow). Histology was evaluated with H&E staining. Scale bar: 100 mm. (**B**-**D**) The villus height, crypt depth, and villus/crypt ratio shown were randomly measured three times in typical images of each sample. (**E**-**F**) The bacterial loads of *S.* Enteritidis in liver and spleen. S.E., *S*. Enteritidis; *n* = 5 per group; data are represented as mean ± SEM. (*p ≤ 0.05, **p ≤ 0.01, ***p ≤ 0.001)

H&E staining of ileal sections in infected mice revealed severe intestinal mucosal damage with loss of intestinal villi; a small amount of intestinal gland necrosis and dissolution in the lamina propria was replaced by hyperplastic connective tissue with punctate lymphocyte infiltration (Fig. 8A). Contrastingly, GCTA reduced the degree of intestinal injury and improved inflammatory exudation (Fig. 8A). Notably, the 100 mg/kg GCTA-treated group exhibited local villus repair, increased intestinal glands, muscle fibers with normal morphology and regular arrangement and no inflammation (Fig. 8A). GCTA treatment also increased the villus length, crypt depth, and villus-to-crypt ratio (VCR) (Fig. 8B-D).

### GCTA inhibited *S.* Enteritidis colonization

The quantification of *S*. Enteritidis colonization was conducted in the liver and spleen of infected mice. Treatment with 100 mg/kg GCTA resulted in a significant decrease in the bacterial load in both the liver and spleen, as observed in Fig. 8E and F, in comparison to mice received no treatment.

## Discussion

This study aimed to assess the potential of GCTA as an alternative to antibiotics, by evaluating its antibacterial properties and mechanisms against *S.* Enteritidis both in vitro and in vivo. *Salmonella* infections pose a significant threat to animal production and human health, and phytochemicals represent a potential alternative treatment strategy. Tannic acid from *Galla chinensis* displays a variety of biological activities including antioxidant, antiviral, and antibacterial activity [15]. Here, we investigated the feasibility of using GCTA as a naturally derived antimicrobial agent, explored its multifaceted mechanisms of action and highlighted its potential for combating *Salmonella* infections.

Chromatographic fingerprinting is a HPLC-based method used to evaluate the quality and chemical constituents of herbal medicines [27]. Existing research demonstrates that GC is rich in gallotannins, comprising a central glucose core encompassed by multiple gallic acid units [15]. . Fingerprinting revealed that gallotannins such as methyl gallate, 1,2,3,6-tetra-O-galloyl-β-D-glucose and 1,2,3,4,6-penta-O-galloyl-β-D-glucose are major secondary metabolites. These polyphenolic substances are known to have active pharmacology including antioxidant, antiviral, and antibacterial activity [28].

Research findings indicate that GCTA exhibits potential as an effective antibacterial agent in combating *Salmonella* infections [14]. The MIC and MBC of GCTA against S. Enteritidis ATCC13076 were 0.72 mg/mL and 1.43 mg/mL respectively, proving that GCTA had strong antibacterial effects even at low concentrations. Further testing revealed that GCTA reduced bacterial growth in a dose- and time-dependent manner. Shu et al. [20] reported the MIC of tannic acid to *S*. Typhimurium was over 2.048 g/mL, nearly three times greater than that of GCTA in this study. This could reflect a synergistic effect of the variety of gallotannins in GCTA.

*Salmonella* relies on specific invasion mechanisms and virulence factors for successful infection and survival in vivo [23]. Phenolic compounds such as tannic acid [20], paeonol [29] and pyrogallol [30] have been shown to inhibit bacterial adhesion and invasion of both RAW264.7 macrophages and epithelial cells in vitro. GCTA had a similar inhibitory effect, decreasing the adhesion and invasion of *S*. Enteritidis to RAW264.7 macrophages and the adhesion of *S*. Enteritidis to Caco-2 epithelial cells. This differ impact on these cells is likely attributed to their distinct functions; macrophages act as immune defenders employing phagocytosis, while epithelial cells are primary targets for bacterial adhesion [31]. Despite GCTA’s modest effect on the bacterial adhesion of epithelial cells, its potential to modulatd immune responses suggests therapeutic significance in limiting *Salmonella* infection.

The TMT-labeled proteomic analysis revealed that GCTA influenced *S.* Enteritidis through several different pathways including DNA replication, repair, and genomic stability, cell wall biogenesis, and lipid metabolism. Notably, GCTA treatment downregulated key enzymes involved in DNA replication and maintenance, such as DNA polymerase and helicase. This downregulation could impact the accuracy and fidelity of bacterial DNA replication, potentially leading to genomic instability and impairment of the ability to cope with environmental stressors; this could influence the development of drug resistance [32]. Furthermore, we observed a downregulation of mismatch repair and homologous recombination pathways, suggesting that GCTA may hinder the ability to repair DNA damage [33].

Folate biosynthesis, an essential process for one-carbon metabolism, was also significantly affected by GCTA treatment. Folate serves as a critical one-carbon carrier in the cell and contributes to redox balance and reductive biosynthesis. Folate is also a strong antioxidant, contributing significantly to cellular redox balance and reductive biosynthesis [34]. Disruption of folate biosynthesis could result in genomic instability, base mismatches, DNA breakage, and chromosome aberrations [35], further supporting a role for GCTA in disrupting *Salmonella* genomic stability.

For microorganisms, the membrane and cell wall are critical barriers to environmental stressors. Destruction of the cell membrane, one of the major targets of phenolic acids, could cause leakage of intracellular contents including sugars and DNA [36]. Accordingly, the alterations we have observed in proteins involved in cell wall, membrane, and envelope biogenesis suggest that GCTA caused membrane damage and compromised cell integrity, although this requires further experimental evidence.

GCTA impacted lipid metabolism, which is crucial for energy storage, cell structure, and biofilm formation [37]. Previous studies have confirmed that phenolic acid alters lipid modification and transport in *Escherichia coli*, reducing lipid and fatty acid oxidation and interrupting energy metabolism [38]. The downregulation of proteins associated with glycerophospholipid metabolism and lipopolysaccharide biosynthesis supports a role of GCTA in disrupting lipid metabolism. Importantly, targeting lipopolysaccharide biosynthesis has proven effective against most gram-negative bacteria, making it an appealing therapeutic approach [39]. Additionally, the membrane damage observed by TEM highlights the potential of GCTA to disrupt cell integrity and may contribute to its antibacterial properties. Thus, GCTA might exert its antimicrobial effects in *Salmonella* by targeting multiple pathways including DNA replication, repair, genomic stability, cell wall biogenesis, and lipid metabolism. The present findings shed light on the molecular mechanisms underlying the antimicrobial efficacy of GCTA and provide valuable insights for its potential application as an alternative treatment against *Salmonella*.

Safety and efficacy are fundamental in the provision of herbal medicines for health care [40]. UDP is an appropriate method to evaluate herbal medicine, as it offers advantages such as a shorter experiment duration, cost-effectiveness, and reproducible outcomes [41]. We found that the LD_50_ of GCTA in mice was 4100 mg/kg with a single oral dose. Contrastingly, a single-dose LD_50_ in rats exceeded 5000 mg/kg when administered in solution [42]. The reason for this discrepancy may be that the quantity of Gallic acid in GCTA used in this study reached 716.8 mg/g, higher than achieved in *Galla chinensis* solution. Safety and efficacy are fundamental in the provision of herbal medicines for health care [40]. UDP is an appropriate method to evaluate herbal medicine, as it offers advantages such as a shorter experiment duration, cost-effectiveness, and reproducible outcomes [41]. We found that the LD_50_ of GCTA in mice was 4100 mg/kg with a single oral dose. Contrastingly, a single-dose LD_50_ in rats exceeded 5000 mg/kg when administered in solution [42]. The reason for this discrepancy may be that the quantity of Gallic acid in GCTA used in this study reached 716.8 mg/g, higher than achieved in *Galla chinensis* solution. Of particular interest was the observation that administering GCTA at the aforementioned dose led to a divergence in outcomes, with both survival and mortality observed. Analogous findings reported by Zhang et al. [43], notably with nicotine doses, suggest an increased variability in outcomes near the LD_50_ threshold. Such variability underscores the intricate nature of herbal medicine responses and underscores the importance of meticulous dosage considerations, particularly around inflection points of dose-response curves. As for hydrolysable tannins, these are considered safe in animals when used as a feed additive up to 15 mg/kg [44]. These guidelines were used to determine that the therapeutic dose was safe for the infected mice in the present study.

*Salmonella* infection triggers the release of cytokines such as IL-6, IL-1β and TNF-α, resulting in systemic inflammatory stress [45]. In this study, IL-1β, IL-6 and TNF-α serum levels dropped significantly in infected mice following GCTA treatment. Although there was no significant change in IL-10, it showed an upward trend after infection which was reduced by GCTA, consistent with previous results [46]. These findings suggest that GCTA modulates the inflammatory response and may create an anti-inflammatory environment, which could be further supported by further study.

*S.* Enteritidis typically infects the gut through contaminated food or water, and colonization leads to host enteric disease [47]. Tannic acid has been widely studied for its ability to protect intestinal health [48]. We found that *Salmonella* challenge damaged the intestinal epithelial villi and crypts in the ileum of mice, consistent with previous reports [49]. Interestingly, GCTA treatment significantly improved the VCR by increasing villus height and decreasing crypt depth in the ileum of mice, effectively alleviating the damage caused by *S.* Enteritidis infection. High VCR is considered a biomarker of intestinal mucosal turnover, indicating strong digestion and absorption capacity [50].

Disruption of the intestinal epithelial structure facilitates the spread of *S.* Enteritidis to the liver and spleen [51]. GCTA treatment resulted in a significant reduction in bacterial loads within the liver and spleen, which exhibited a positive correlation with the enhanced integrity of the intestinal epithelium. This suggests that GCTA treatment alleviated the spread of *Salmonella* infection; this could be associated with regulating the inflammatory response, improving intestinal histopathological alterations and lowering the bacterial load in the liver and spleen in infected mice Thus, GCTA could represent a potential natural antimicrobial agent for the control of *Salmonella* infection.

## Conclusion

We demonstrated that GCTA had significant antibacterial and anti-infective activities against *S*. Enteritidis. These properties may be related to the ability of GCTA to inhibit invasion, pathways including genomic repair and stability, cell wall biogenesis and lipid metabolism, and alter the inflammatory response, which was reflected by accompanying intestinal histopathological changes. This suggests GCTA could be developed as an alternative or supplemental agent to manage *Salmonella* infections. This study offered valuable insights into certain molecular mechanisms that underlie the antibacterial effects of GCTA. However, it is important to note that there are additional mechanisms at play that warrant further investigation.

### Electronic supplementary material

Below is the link to the electronic supplementary material.

Supplementary Material 1

## Data Availability

The datasets generated for this study are available on request to the corresponding author.
