# Supplementary material for: Battling Salmonella enteritidis infections: integrating proteomics and in vivo assessment of Galla Chinensis tannic acid
Source: BMC Vet Res. 2024 May 7;20:179. doi: 10.1186/s12917-024-04036-5 (PMC11075308; doi:10.1186/s12917-024-04036-5)
Supplement: Supplementary file 1 — Supplementary Material 1 [file 12917_2024_4036_MOESM1_ESM.docx]

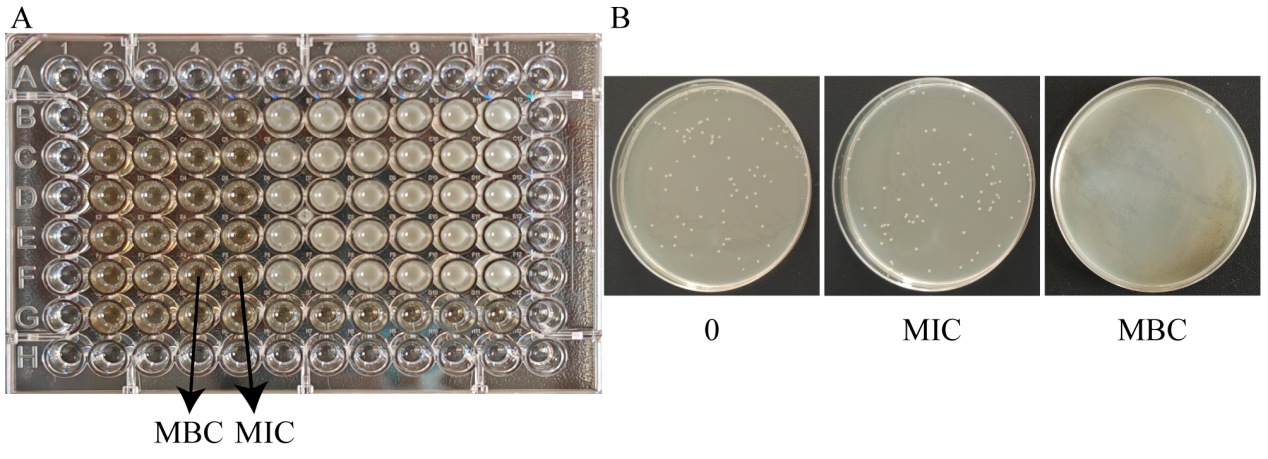


Figure S1 The MIC and MBC of GCTA against *S.* Enteritidis ATCC13076. (A) The photograph of the determination of MIC and MBC of GCTA against *S.* Enteritidis using the broth microdilution method. Rows B-F represent replicate wells, while row G serves as the negative control without bacteria. Columns 2-11 indicate concentrations of 44.8, 22.4, 11.2, 5.6, 2.8, 1.4, 0.7, 0.35, 0.175, and 0 mg/mL GCTA. (B) LB agar plate shows the counting of bacterial colonies; left: bacterial count before incubation, middle: bacterial count at MIC concentration, right: bacterial count at MBC concentration.

Table S1 Differential expression of proteins involved in genomic stability identified by TMT-labeled quantitative proteomics in *S.* Enteritidis

| **Description** | **S.E.** | **S.E.+ GCTA** | **S.E.+ GCTA/S.E. ratio** | ***P*-value** |
| --- | --- | --- | --- | --- |
| DNA helicase | 1.54E+05 | 8.43E+04 | 5.49E-01 | 4.37E-02 |
| RecBCD enzyme subunit RecC | 7.53E+04 | 4.72E+04 | 6.27E-01 | 2.31E-02 |
| RecBCD enzyme subunit RecB | 1.45E+05 | 9.71E+04 | 6.68E-01 | 3.83E-02 |
| DNA helicase (Fragment) | 5.59E+04 | 3.95E+04 | 7.07E-01 | 3.03E-02 |
| ATP-dependent DNA helicase RecG | 3.40E+04 | 2.44E+04 | 7.18E-01 | 2.48E-02 |
| DNA mismatch repair protein MutH | 3.28E+04 | 1.79E+04 | 5.47E-01 | 2.12E-02 |
| DNA mismatch repair protein MutS | 5.96E+05 | 3.31E+05 | 5.55E-01 | 3.87E-02 |
| UvrABC system protein C | 3.31E+04 | 2.31E+04 | 6.98E-01 | 3.33E-02 |
| DNA polymerase III subunit delta | 5.75E+04 | 3.54E+04 | 6.16E-01 | 5.90E-03 |
| Beta sliding clamp | 3.46E+05 | 2.50E+05 | 7.22E-01 | 3.84E-02 |
| DNA polymerase III subunit alpha | 2.06E+05 | 1.58E+05 | 7.68E-01 | 1.89E-02 |
| Transcription-repair-coupling factor | 5.97E+05 | 3.49E+05 | 5.84E-01 | 1.76E-02 |
| DNA repair protein RadA | 1.72E+05 | 1.09E+05 | 6.31E-01 | 4.03E-02 |
| Exodeoxyribonuclease I | 2.06E+05 | 1.15E+05 | 5.59E-01 | 3.09E-02 |
| Primosomal replication protein N | 2.04E+03 | 3.18E+03 | 1.56E+00 | 3.08E-02 |
| Dihydrofolate synthase/folylpolyglutamate synthase | 1.01E+05 | 4.51E+04 | 4.48E-01 | 1.54E-02 |
| Aminodeoxychorismate synthase | 1.30E+05 | 7.19E+04 | 5.51E-01 | 1.32E-02 |
| 6-carboxy-5,6,7,8-tetrahydropterin synthase (Fragment) | 4.37E+03 | 8.40E+03 | 1.92E+00 | 1.07E-02 |
| 7-cyano-7-deazaguanine synthase | 5.59E+04 | 7.65E+04 | 1.37E+00 | 3.39E-02 |
| 7-carboxy-7-deazaguanine synthase | 5.31E+04 | 3.87E+04 | 7.29E-01 | 4.72E-02 |
| Molybdopterin molybdenumtransferase (Fragment) | 9.91E+04 | 7.29E+04 | 7.36E-01 | 2.25E-02 |
| NADPH-dependent 7-cyano-7-deazaguanine reductase | 2.36E+05 | 1.78E+05 | 7.52E-01 | 2.80E-02 |

Table S2 Differential expression of proteins involved in cell wall/membrane/envelope biogenesis identified by TMT-labeled quantitative proteomics in S. Enteritidis

| **Description** | **S.E.** | **S.E.+ GCTA** | **S.E.+ GCTA/S.E. ratio** | ***P*-value** |
| --- | --- | --- | --- | --- |
| Linear amide C-N hydrolase (Fragment) | 1.23E+05 | 8.79E+05 | 7.16E+00 | 2.08E-03 |
| Glycosyl transferase family 2 | 3.17E+05 | 1.11E+05 | 3.52E-01 | 9.09E-03 |
| Multidrug resistance protein MdtA | 1.20E+05 | 8.43E+05 | 7.00E+00 | 3.56E-03 |
| Mannose-1-phosphate guanylyltransferase | 2.61E+05 | 1.02E+05 | 3.91E-01 | 1.70E-02 |
| Lipopolysaccharide biosynthesis protein LPS:glycosyltransferase | 4.86E+04 | 2.02E+04 | 4.15E-01 | 3.27E-02 |
| Mechanosensitive ion channel family protein | 4.66E+05 | 1.99E+05 | 4.27E-01 | 4.91E-02 |
| ADP-heptose--LPS heptosyltransferase RfaF | 1.36E+05 | 5.82E+04 | 4.28E-01 | 4.09E-02 |
| Putative polysaccharide export protein (Fragment) | 4.83E+04 | 1.79E+05 | 3.71E+00 | 2.02E-03 |
| YjbH domain-containing protein | 2.86E+04 | 9.63E+04 | 3.37E+00 | 1.17E-02 |
| Lipopolysaccharide core heptosyltransferase RfaQ | 1.82E+05 | 8.49E+04 | 4.67E-01 | 3.50E-02 |
| Recombinase | 4.24E+04 | 1.23E+05 | 2.90E+00 | 3.93E-02 |
| Glycosyl transferases group 1 | 3.57E+05 | 1.83E+05 | 5.14E-01 | 3.27E-02 |
| N-acetylmuramoyl-L-alanine amidase | 5.82E+03 | 1.18E+04 | 2.02E+00 | 2.50E-02 |
| UDP-N-acetyl-D-mannosamine dehydrogenase | 4.68E+05 | 2.50E+05 | 5.35E-01 | 1.67E-02 |
| UPF0194 membrane protein YbhG | 1.73E+05 | 3.10E+05 | 1.79E+00 | 1.07E-02 |
| Phosphoglycerol transferase I | 6.16E+05 | 3.46E+05 | 5.61E-01 | 2.66E-02 |
| Autotransporter outer membrane beta-barrel domain-containing protein | 6.56E+03 | 1.16E+04 | 1.77E+00 | 1.91E-02 |
| Penicillin-binding protein 1A | 4.63E+05 | 2.63E+05 | 5.68E-01 | 3.93E-03 |
| Arabinose 5-phosphate isomerase | 3.99E+05 | 2.30E+05 | 5.77E-01 | 3.05E-02 |
| Peptidoglycan D,D-transpeptidase MrdA | 4.74E+04 | 2.76E+04 | 5.82E-01 | 2.38E-03 |
| Lipid A biosynthesis lauroyltransferase | 2.92E+04 | 1.73E+04 | 5.93E-01 | 1.66E-02 |
| dTDP-glucose 4,6-dehydratase | 3.68E+05 | 2.21E+05 | 6.02E-01 | 4.70E-02 |
| Lipopolysaccharide 1,6-galactosyltransferase (Fragment) | 5.64E+03 | 9.06E+03 | 1.60E+00 | 2.14E-02 |
| Murein DD-endopeptidase MepM | 4.21E+04 | 6.75E+04 | 1.60E+00 | 2.93E-02 |
| UDP-N-acetylglucosamine--N-acetylmuramyl-(pentapeptide) pyrophosphoryl-undecaprenol N-acetylglucosamine transferase | 1.95E+05 | 1.29E+05 | 6.59E-01 | 1.80E-02 |
| Peptidoglycan lytic exotransglycosylase | 7.40E+05 | 4.88E+05 | 6.60E-01 | 1.64E-02 |
| Glucarate dehydratase | 3.01E+05 | 2.03E+05 | 6.75E-01 | 3.82E-02 |
| UDP-N-acetylmuramoyl-L-alanyl-D-glutamate--2,6-diaminopimelate ligase | 3.47E+05 | 2.39E+05 | 6.88E-01 | 4.48E-02 |
| Glutamate racemase | 2.66E+05 | 1.90E+05 | 7.14E-01 | 4.16E-02 |
| LPS O-antigen length regulator | 1.25E+05 | 9.12E+04 | 7.32E-01 | 1.42E-02 |
| Undecaprenyl-phosphate galactose phosphotransferase | 5.31E+05 | 3.89E+05 | 7.33E-01 | 4.27E-03 |
| Lipoprotein | 7.58E+04 | 1.03E+05 | 1.36E+00 | 5.00E-02 |
| dTDP-glucose 4,6-dehydratase | 1.04E+05 | 7.81E+04 | 7.54E-01 | 2.30E-02 |
| Membrane-bound lytic murein transglycosylase A | 2.62E+05 | 1.98E+05 | 7.56E-01 | 1.00E-03 |
| Molecular chaperone FimC | 4.82E+04 | 6.26E+04 | 1.30E+00 | 4.30E-02 |
| Penicillin-binding protein activator LpoB | 4.52E+04 | 5.86E+04 | 1.30E+00 | 4.10E-02 |

Table S3 Differential expression of proteins involved in lipid metabolism identified by TMT-labeled quantitative proteomics in S. Enteritidis

| **Description** | **S.E.** | **S.E.+ GCTA** | **S.E.+ GCTA/S.E. ratio** | ***P*-value** |
| --- | --- | --- | --- | --- |
| Fatty acid oxidation complex subunit alpha | 1.09E+06 | 3.34E+05 | 3.05E-01 | 4.55E-04 |
| CDP-diacylglycerol--serine O-phosphatidyltransferase | 3.96E+05 | 1.62E+05 | 4.10E-01 | 1.46E-02 |
| 3-ketoacyl-CoA thiolase | 2.58E+05 | 1.24E+05 | 4.79E-01 | 1.90E-02 |
| Fatty acid oxidation complex subunit alpha | 1.82E+05 | 8.87E+04 | 4.88E-01 | 9.44E-03 |
| Glycerol-3-phosphate acyltransferase | 5.19E+05 | 2.62E+05 | 5.05E-01 | 5.74E-03 |
| Long-chain-fatty-acid--CoA ligase FadD | 4.99E+04 | 2.79E+04 | 5.59E-01 | 3.76E-02 |
| CDP-diacylglycerol pyrophosphatase (Fragment) | 6.04E+05 | 3.49E+05 | 5.77E-01 | 3.42E-02 |
| Cardiolipin synthase A | 5.26E+04 | 3.07E+04 | 5.84E-01 | 2.68E-02 |
| 2,3-dihydro-2,3-dihydroxybenzoate dehydrogenase | 7.40E+05 | 1.20E+06 | 1.62E+00 | 4.81E-02 |
| Bifunctional protein Aas | 5.09E+05 | 3.67E+05 | 7.21E-01 | 3.02E-02 |
| 1-deoxy-D-xylulose 5-phosphate reductoisomerase | 1.40E+05 | 1.02E+05 | 7.33E-01 | 4.34E-02 |
| Uncharacterized Nudix hydrolase YfcD | 1.08E+05 | 1.46E+05 | 1.36E+00 | 2.75E-02 |
| 2-C-methyl-D-erythritol 4-phosphate cytidylyltransferase | 7.12E+04 | 8.83E+04 | 1.24E+00 | 3.16E-03 |
| Lipid kinase, YegS/Rv2252/BmrU family | 5.68E+04 | 1.07E+05 | 1.88E+00 | 9.24E-03 |
| NAD(P)-binding protein | 7.96E+03 | 5.80E+03 | 7.28E-01 | 2.47E-02 |
| Alcohol dehydrogenase AdhP | 4.64E+05 | 9.28E+05 | 2.00E+00 | 4.35E-02 |

Table S4 Differential expression of proteins involved in glycerophospholipid metabolism identified by TMT-labeled quantitative proteomics in S. Enteritidis

| **Description** | **S.E.** | **S.E.+ GCTA** | **S.E.+ GCTA/S.E. ratio** | ***P*-value** |
| --- | --- | --- | --- | --- |
| CDP-diacylglycerol--serine O-phosphatidyltransferase | 3.96E+05 | 1.62E+05 | 4.10E-01 | 1.46E-02 |
| Glycerol-3-phosphate acyltransferase | 5.19E+05 | 2.62E+05 | 5.05E-01 | 5.74E-03 |
| Glycerol-3-phosphate dehydrogenase | 2.39E+06 | 1.26E+06 | 5.29E-01 | 2.09E-03 |
| Lipid kinase, YegS/Rv2252/BmrU family | 5.68E+04 | 1.07E+05 | 1.88E+00 | 9.24E-03 |
| CDP-diacylglycerol pyrophosphatase (Fragment) | 6.04E+05 | 3.49E+05 | 5.77E-01 | 3.42E-02 |
| Cardiolipin synthase A | 5.26E+04 | 3.07E+04 | 5.84E-01 | 2.68E-02 |
| Bifunctional protein Aas | 5.09E+05 | 3.67E+05 | 7.21E-01 | 3.02E-02 |

Table S5 Differential expression of proteins involved in lipopolysaccharide biosynthesis identified by TMT-labeled quantitative proteomics in S. Enteritidis

| **Description** | **S.E.** | **S.E.+ GCTA** | **S.E.+ GCTA/S.E. ratio** | ***P*-value** |
| --- | --- | --- | --- | --- |
| Lipopolysaccharide biosynthesis protein LPS:glycosyltransferase | 4.86E+04 | 2.02E+04 | 4.15E-01 | 3.27E-02 |
| ADP-heptose--LPS heptosyltransferase RfaF | 1.36E+05 | 5.82E+04 | 4.28E-01 | 4.09E-02 |
| Lipopolysaccharide core heptosyltransferase RfaQ | 1.82E+05 | 8.49E+04 | 4.67E-01 | 3.50E-02 |
| Glycosyl transferases group 1 | 3.57E+05 | 1.83E+05 | 5.14E-01 | 3.27E-02 |
| Arabinose 5-phosphate isomerase | 3.99E+05 | 2.30E+05 | 5.77E-01 | 3.05E-02 |
| Lipid A biosynthesis lauroyltransferase | 2.92E+04 | 1.73E+04 | 5.93E-01 | 1.66E-02 |
| Lipopolysaccharide 1,6-galactosyltransferase (Fragment) | 5.64E+03 | 9.06E+03 | 1.60E+00 | 2.14E-02 |
| Heptose kinase | 9.45E+04 | 6.32E+04 | 6.68E-01 | 4.45E-02 |
